# Smartphone activated community first responders’ experiences of out-of-hospital cardiac arrests alerts, a qualitative study

**DOI:** 10.1016/j.resplu.2022.100246

**Published:** 2022-05-18

**Authors:** Marie-Louise Södersved Källestedt, Harald Lindén, Petronella Bjurling-Sjöberg

**Affiliations:** a Clinical Skills Center, Centre for Clinical Research, Uppsala University, Region Västmanland, Västerås, Mälardalen University, Sweden; b Department of Patient Safety Region Sörmland, Eskilstuna, Sweden; c Department of Public Health and Caring Sciences, Caring Science, Uppsala University, Uppsala, Sweden & Centre for Clinical Research Sörmland, Uppsala University, Eskilstuna, Sweden

**Keywords:** Out-of-hospital cardiac arrest, Cardiopulmonary resuscitation, Volunteers, Smartphone, AED, Automated external defibrillator, CPR, Cardiopulmonary resuscitation, CFRs, Community first responders, OHCA, Out-of-hospital cardiac arrest

## Abstract

**Aim:**

The aim was to illustrate how community first responders perceive out-of-hospital cardiac arrest alerts delivered via smartphone, what support they have and how they cope with potentially distressing experiences.

**Method:**

A qualitative interview study was conducted with a volunteer sample of 14 community first responders in two regions of Sweden. The interviews were transcribed and analysed using thematic analysis with a data-driven inductive approach supported by NVivo 1.3.

**Results:**

The responders’ experiences were illustrated in three main themes, each including several subthemes: 1) *Profound wish to help*, including the sense of importance and sense of emergency; 2) *Facing the situation*, including essential actions performed in collaboration, confidence from training and experience, challenges posed by unforeseen situations and ethical dilemmas, and coping with emotional reactions; and 3) *Potential for improvements*, including technical and communication development, feedback and debriefing, training and social marketing*.*

**Conclusion:**

The community first responders were motivated and eager to help but simultaneously feared the mission and were not always prepared for their own reactions in the emergency when dispatched. Although cardiopulmonary resuscitation training and experience gave them skills that enabled them to act constructively, they faced situations that might be facilitated by improvements in the community first responder system and further training. The responders were proud of their efforts and were good ambassadors for the system. Appreciation of their commitment, better preparation and providing support in the aftermath of an emergency appears to be a good investment in societies’ efforts to bring quick help to distressed persons.

## Introduction

Out-of-hospital cardiac arrest (OHCA) is a significant public health concern, with an annual incidence of 67-170/100 000 inhabitants in European countries,1., 2. and a mean survival rate of eight percent.^3^ Time to cardiopulmonary resuscitation (CPR)^4^ significantly affects the survival rate.5., 6., 7., 8. Bystander CPR performed while waiting for emergency medical services can more than double the 30-day survival rate of people who experience OHCA^9^ and positively affect neurological outcomes.^10^ Using a smartphone-based positioning system to dispatch CPR-trained volunteers, hereafter referred to as community first responders (CFRs), and automated external defibrillators (AEDs) to OHCA situations increases the chance for quick help while waiting for emergency medical services,7., 11. and such systems are increasingly utilized in several countries.12., 13.

Although utilizing CFR systems is recommended in the European Resuscitation Council guidelines^2^ and consensus prevails that such systems saves lives,^14^ these systems are relatively new, and knowledge of the potential side effects of their use on volunteers is scarce. Voluntarily performing CPR requires knowledge,^15^ engagement, and considerations of the consequences of participation in potentially distressing situations.^16^ To provide appropriate opportunities to meet these needs, further knowledge is needed about how the CFR system is experienced by the involved volunteers. The aim of this study was to illustrate how CFRs perceive OHCA alerts, what support they have and how they cope with potentially distressing experiences.

## Methods

### Study design and ethics

This interview study had a qualitative descriptive design with a data-driven inductive approach,17., 18. following the Consolidated Criteria for Reporting Qualitative Research.^19^ The Swedish ethical review authority in Uppsala (Dnr 2019-05780) approved the study. Participants gave informed consent.

### Context

Two mid-sized Swedish regions with both urban and rural areas were included.

In Sweden, AEDs are accessible in some public places, and CPR training is provided through a standardized program that is designed for both lay people and healthcare workers. The program includes lecture and practical training and is standardized for content, teaching material and teaching methodology. The program was originally provided through a cascade principle in which instructor trainers taught instructors who would then teach rescuers, which has allowed efficient dissemination to a large number of people.^20^ In recent years, the standardized CPR training has also been provided as a web program, which has reduced the dependence on instructors.

The included regions have been affiliated with the HeartRunner^21^ CFR system since 2018 (Appendix 1). CFRs who sign up must be a minimum of 18 years old and are recommended to have completed CPR training within the last 12 months. In 2019, CFRs were dispatched through the system in 175 OHCR alerts in the regions. In 2020, the system was periodically paused due to the COVID-19 pandemic.

### Participants and data collection

Qualitative consecutive individual interviews with CFRs who had been dispatched at least once were performed. Informants were recruited in 2019–2020 through a message in the CFR system’s smartphone application (app) that informed them about the study and invited them to schedule an interview. CFRs were included until data saturation was achieved.

A semistructured interview guide was developed by the authors based on their experience and the current literature.14., 22., 23., 24., 25., 26, 27. The guide was pilot tested in two interviews by MLSK and HL and used by all authors (Appendix 2). The opening question was “Can you please tell me about when you were called out as a CFR?” Thereafter, probes were used to cover the following areas: a) training and previous experience in CPR, b) reactions and actions when alerted, and c) thoughts about being a CFR.

Face-to-face or remote interviews (median duration 33 minutes) were performed and audio recorded in 2020 (Table 1). The interviewers (authors) were all experienced registered nurses (two females, one male) without previous relationships with the informants. MLSK and HL were experienced CPR instructor trainers. HL was the administrator of a CFR system. MLSK and PBS (both PhD) were experienced in the interview technique and qualitative analysis.

**Table 1:** Characteristics of the included Smartphone activated community first responders and the interviews.

Characteristics (N = 14)		Value
Gender	Female	4
	Male	10
Age	30–39 years	3
	40–49 years	5
	50–59 years	4
	60–69 years	2
Region	Sörmland	7
	Västmanland	7
Profession	Lay person^1^	8
	Healthcare worker^2^	4
	Rescue professional^3^	2
Last CPR training	≤ 6 months ago	6
	1 year ago	4
	2 years ago	3
	3–5 years ago	1
Number of alerts in the CFR-system	One time	5
	Two to three times	6
	Four to six times	3
Last CFR mission	≤ 6 months ago	5
	7–12 months ago	5
	>12 months ago	3
	Unknown	1
Interview	Face-to-face^4^	9
	Distance^5^	5
Interview minutes	Median (range)	33 (10-45)

CFR; Community First Responder (Smartphone activated).

CPR; Cardiopulmonary Resuscitation.

1 Includes people with CPR training but without any professional healthcare or rescue education.

2 Includes two registered nurses, one nursing teacher and one healthcare concern manager.

3 Includes one police officer and one firefighter.

4 At a place chosen by the informant (the hospital or the informants home).

5 Due to the COVID-19 pandemic distance meeting by telephone/digital video conference.

### Data analysis

**Fig. 1:**
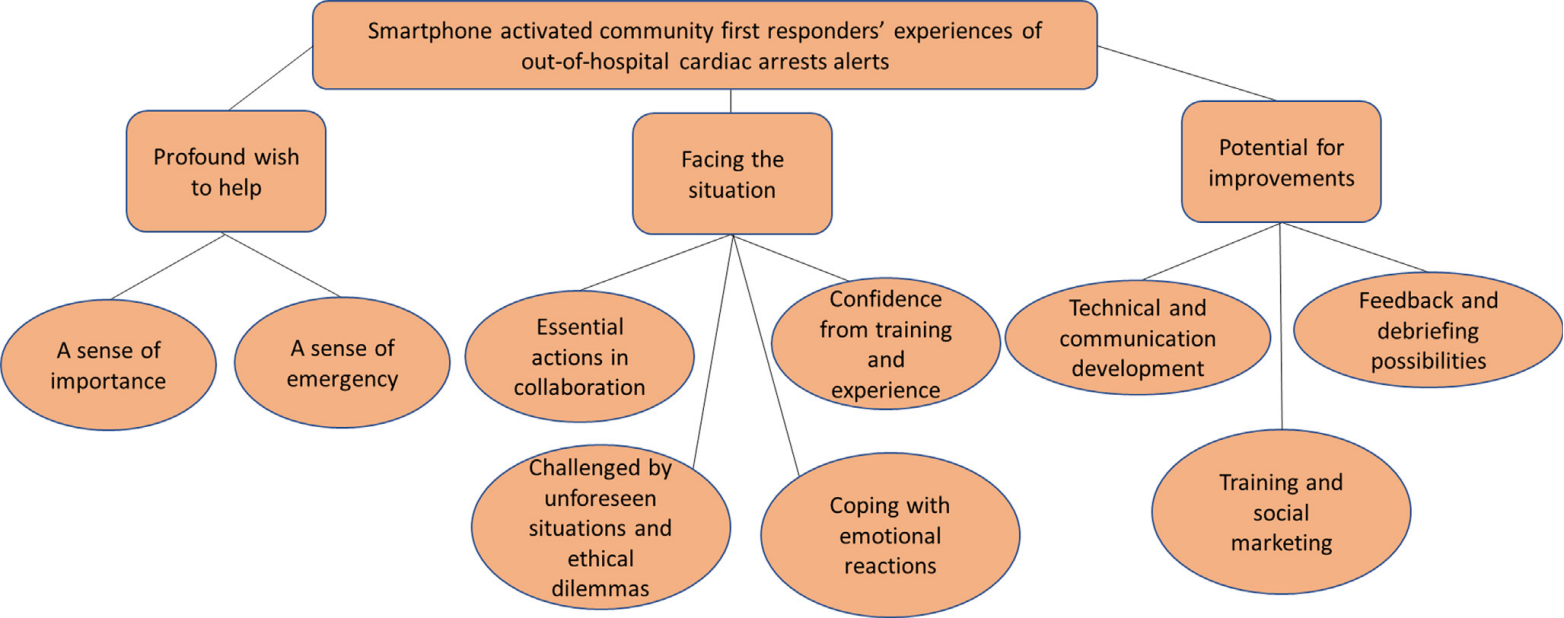
Thematic map of Smartphone activated community first responders’ experience of out-of-hospital cardiac arrests alerts.

The interviews were transcribed and managed in NVivo (release 1.3) using thematic analysis.17., 18. The transcriptions were read repeatedly for familiarization. Significant parts were extracted, coded and organized into sets with similar meanings to create themes. A thematic map (Fig. 1) was generated after refining and checking the themes and subthemes in relation to the extracts and the entire dataset. Two authors (MLSK and PBS) led the analysis. To promote credibility, all authors contributed to the coding and read and discussed the content throughout the analysis process.

## Results

The study included 14 CFRs from the two included regions, with variations in gender, age, profession and experience (Table 1). In total, the CFRs described 31 OHCA alerts in which they had contributed in a variety of roles (Table 2). Based on the thematic analysis, the CFR experiences were illustrated in three main themes: 1) *Profound wish to help*, 2) *Facing the situation*, and 3) *Potential for improvements*. Fig. 1 illustrates the themes and subthemes in a thematic map. Examples of the codes and quotations are presented in Table 3.

**Table 2:** The Smartphone activated community first responders’ role at the resuscitation attempt and response on the instructions regarding defibrillator in each alert.

Characteristics		Value
Role at the resuscitation attempt	Hands-on CPR^1^	6
	Other action^2^	6
	Reached the site but was not needed	11
	Unable to reach the site	2
	Declined the alarm	6
Instructed to bring a AED^3^	No	17
	Yes	8
	– Brought an AED	2
	– Tried to, but did not reach any	1
	– Did not follow the instruction^4^	5
Total number of experienced OHCR alerts in the CFR-system		31

AED; Automated External Defibrillator.

CFR; Community First Responder (Smartphone activated).

CPR; Cardiopulmonary Resuscitation.

OHCA; Out-of-hospital cardiac arrest.

1 Chest compressions, mouth-mouth ventilation and/or use of AED.

2 Other actions included: Monitored the distressed person, helpful to emergency professionals, supporting the victim’s relatives and others at the mission, facilitated by hindering unnecessary people from entering the site or making passageway for the Emergency Medical Service.

3 Includes only the 25 alerts that were accepted.

4 The CFR perceived it more expedient to immediately go to the site of the OHCA since the assigned AED was located too far away or in a locked place.

**Table 3:** Main theme sub themes, example of codes and quotations from the Smartphone activated community first responders.

*Main theme*		
Sub themes	Example of codes	Exampel of quotations (translated from Swedish)
*Profound wish to help*		
A sense of importance	Motivated by fellow humanity A sense of obligation to society Personal emotional motive Eager to help Fear for what to expect Responsibility of up-to-date skill and preperation Frustration of technical/practical hinders A sense of guilt when unable to accept alert Proud when completed the mission Inspire others to volunteer	– I feel that as I have the knowledge I want to contribute/…/if you want to receive help you have to be willing to give help as well (Informant 12, healtcare worker). – It is like a hate-and-love feeling/… / a good feeling to be involved in saving a life, on the other hand you can end-up with a lost life too, which I think may make people hesitate (Informant 4, lay person). – On may own initiative, drive around to check were the AED are located and how I access them (Informant 14, rescue professional). – When I recected I thought, what happens now? Was I the only one who got this alert? Was I so selfish that I rejected, and a man died? That was the start of my vaccation. (Informant 11, lay person).
A sense of emergency	Surprised in everyday life An adrenalin rush Getting a goal-oriented focus Assess the situation and make own decisions Engaging family/friends for practical support Everything happened so quickly Long time waiting for the ambulance Surrealistic feeling	– I just went into a different mood; it was really amacing, that a person can have such strength (Informant 2, lay person). – I was ordered to the nearest AED/… / but I chosed not to go there because it had been a significant detour /… / I could start at once instead, so I made that priority (Informant 7, lay person). – When I arrived, it was like surrealistic, the street was empty, the door was open, and when I was inside the man called from upstairs: - It’s up here. And he performed some kind of CPR. (Informant 8, healtcare worker)
*Facing the situation*		
Essential actons in collaboration	Collaborate in resuscitation with bystanders Support from dispatch service Inform and assist healthcare professionals Professional response from EMS Demonstrate knowledge to be of assistance Difficulty to get other CFR to back-of Missunderstod as healthcare worker or family member Missunderstod as family member	– Just when I was about to start another person arrived. I just told him to start mouth-to-mouth ventilation and that I was going to do compressions. Then we started. He was also very well-structured (Informant 5, healtcare worker). – When I arrived there were four persons, it just worked straight-up, no discussion about who should lead and do this and that, we were almost like military (Informant 11, lay person) – He [the husband] did not understand that I was a CFR, he thougt I was from the emergency medical service (Informant 8, healtcare worker).
Confidence from training and experience	Regularly CPR training helpful Being a CPR instructor give confidence in skills Experience of resuscitation in healthcare give confidence CFR experiences facilite prepardness Mental preparedness from other emergencies Less confident than in the professional role	– When you go to the CPR training you think -what will this help? But it actually did, you knew exactly how to act, because you had tried it (Informant 11, lay person). – A hostage situation /… / soo I have experience from taking care of people in that stress (Informant 6, lay person). – When you are in green clothes [emergency medical service work clothes], you get information and know what you are on your way to. In these situations you are more prepared (Informant 1, healtcare worker).
Challenged by unforeseen situations and ethical dilemmas	Issues to find/access AED and the victim Unforessen people on site Unexpected victim (child/other needs) Exposed to risk of contagion Very old victim raise ethical doubts Strange to touch the body of a stranger Strange to be in someone’s home Unsure about how to handle victim’s family	– It was an early Sunday morning, so I did not have access to the AED (Informant 14, rescue professional). – I felt recistance doing mouth-to-mouth ventilation, she had vomited and was severely cyanotic, but I did it anyway. I will never do it again. I will make sure to use the contact protecton next time (Informant 8, healtcare worker). – He was very old. I almost felt guilt, I felt like; can we please let him be? But that was not my decision to take (Informant 3, rescue professional). – It has gnawed a little in me, should I go there and call on the door and express my condolianses or should I just let go? (Informant 2, lay person).
Coping emotional reactions	Connection based on joint experience Being unaffected Existential thoughts Emotional distress Talk with family/friends afterwards Reflektion with CPR instructor Debriefing through employment in healthcare	– The only relief has been that there were no youger persons. That I know is harder to manage (Informant 13, lay person). – I was thinking about it all day: How did that person get along? I Google and checked the newspaper… (Informant 11, lay person). – I had a huge need to just talk and kind of repeating in may head what had happened, how it was (Informant 8, healtcare worker).
*Potential for improvements*		
Technical and communication development	Directed only to accessable AED Precice information of OHCA place and accessability, e.g. access code Possibility for the dispatch service to continusly add information Additional information of the victim Information about other CFR alerted Possibility to confirm that you are at the site Metronome for compression rate Opportunity to communicate with the dispatch service	– I was first on the scence and was searching for the right house (Informant 3, rescue professional). – If the message said something about if it is a child or somthing, to quickly see and be better prepered when running (Informant 5, healthcare worker). – To see in the app how many responders, to know that someone else also will come, becaus CPR is quite exhausting. And, I would like to confirm in the app that I am at the site, like the emergency medical service do, and automaticly be conected to talk with the disptch service to get support (Informant 8, lay person).
Feedback and debriefing possibility	Whish of personal progress Feedback from the resuscitation to learn Feedback of outcome for emotional comfort Offer debriefing possibility Offer contact for emotional support Ask for family preferens of contact Opportunity to connect family and CFR	– You would develop much more if you could bypass confidentiality within the team at site. If everyone were informed about the outcome it would be a possibility to learn. (Informant 1, healthcare worker). – You could have some kind of debriefing, a centralized function or a telephone call, I think that may help many that been exposed for this for the first time (Informant 13, lay person). – I want to tell her how it was /…/ to make her calm and let her know who rushed into her home (Informant 2, lay person).
Training and social marketing	Instruction video in the application Pop-up reminder for refresher training Certificate of CPR training visible in the app Companies could sponsor with training CFR-system information in all CPR training programs Coordinated campainges by the municipinary Inspiering experience and knowledge exchange by CFR meetings	– The municipanities could do more to disseminate this/…seek collaborators/…/my company could contribute with training instructurs and equipment if the municpinary provided premises and marketing (Informant 1, healtcare worker). – A mingle with a cup of coffe/…/sharing experiences and such/…/ it might also bee a token of appriciation (Informant 9, lay person).

AED; Automated External Defibrillator, CFR; Community First Responder (Smartphone-based activated), CPR; Cardiopulmonary resuscitation, OHCA; Out-of-hospital cardiac arrest.

### Profound wish to help

The Profound wish to help theme was expressed in the subthemes *A sense of importance* and *A sense of emergency*.

The CFRs were motivated and eager to help but simultaneously feared the important mission. They prepared themselves mentally and with training, as an informant expressed: “I can think, when I am in the city; if this person collapses, I ask that person to call 112 and I start with this… It’s like I think ahead, so my brain is prepared…” (Informant 8, healthcare worker).

After completing the mission, the responders felt proud and acted as ambassadors to inspire others to sign up as CFRs. An inability to respond or technical issues generated feelings of guilt, disappointment and frustration, and recurring thoughts of the victim.

Several CFRs described the alert as a surprise, and the sense of emergency caused an adrenalin rush, creating a goal-oriented focus. They assessed the possibility of arriving before the emergency medical services, whether to first retrieve an AED and whether they were needed at the site. Several responders involved family or friends to provide practical support when the alert was raised, e.g., providing a ride. The emergency also resulted in a temporarily changed perception of reality, with an altered perception of time and a surrealistic feeling. Additionally, in the aftermath, some of the responders were affected by the adrenalin and perceived the situation as unrealistic.

### Facing the situation

Facing the situation included the subthemes *Essential actions in collaboration, Confidence from training and experience, Challenges posed by unforeseen situations and ethical dilemmas,* and *Coping with emotional reactions*.

On site, the CFRs performed different essential actions (Table 2) in collaboration with bystanders and healthcare workers. The CFRs had experiences of taking command and following orders. Although there were some misunderstandings and issues regarding roles, the interactions were most often perceived as well functioning.

The completion of CPR training enabled confidence, and previous experience with resuscitation was perceived to facilitate preparedness for the next mission. Additionally, experiences of other types of emergency situations were perceived to facilitate preparedness and the ability to perform proper actions. The CFRs who were healthcare workers highlighted that they were less confident in their role as a CFR than in their professional role.

During the mission, the CFRs were challenged by unforeseen situations; e.g., they faced practical issues when they did not gain access to the appointed AED or received incomplete information about the victim’s location. They were unprepared for victims with needs other than CPR and for child victims. Ethical dilemmas occurred regarding the performance of CPR on an elderly victim, the sensation of touching a stranger’s body and the risk of contagion. Being in someone else’s home and dealing with the victim’s family were additional challenges, both during resuscitation and afterwards, as expressed by an informant: “…the thoughts appeared in nightmares; he was such a nice old man, and it was Christmas… I just stepped into their home… That I was not prepared for…” (Informant 2, lay person).

After completing the mission, the responders felt connected to each other based on the joint experience but had no further contact with the resuscitation fellows. In the aftermath, some CFRs were unaffected by the event, but thinking of the victim and the victim’s family, existential thoughts and even nightmares were described, especially by lay responders. To cope, they reached out for emotional support from family, friends, CPR instructors or work colleagues, all of which were perceived as helpful.

### Potential for improvements

The subthemes of Potential for improvements included *Technical and communication development, Feedback and debriefing possibilities,* and *Training and social marketing.*

The CFRs suggested more precise instructions in the app to easily find and access the AED and the victim. Additional information about the victim and other CFRs were requested to increase preparedness and technical and communication support through the app.

To help with efficiency in future resuscitations, the CFRs wanted to develop in their role. Although aware of the confidentiality issue, they requested feedback about the outcome of the emergency to learn and to move forward emotionally, as expressed by an informant: “It was strange that we didn’t have the opportunity to talk with someone that evening…” (Informant 5, healthcare worker). Possibilities for debriefing and receiving emotional support through the app, as well as opportunities for the victim’s family to get in touch with the CFR, were suggested.

To be better prepared for different challenges, information on other health conditions as well as the CFR system was requested to be included in the CPR training programs. To facilitate development, show appreciation and attract more volunteers, better training opportunities, educational meetings for CFRs, and social events and marketing to disseminate knowledge of the CFR system were suggested. Box 1 lists 20 action points to optimize the preparation and conditions for the rescuers.

Box 1 **Action points to include in the CPR training program and/or in the CFR system to optimize the preparation and conditions for the rescuers.**

**Action points for each step of the mission**

Prior to an alert

• Provide CPR training opportunities
• Provide educational meetings and social events for CFRs
• Encourage CFRs to checking nearby AEDs
• Encourage CFRs to train with the test alert in the app
• Provide possibilities for CFRs who are healthcare workers to discuss their different roles as CFRs with minimal information and material

Getting the alert

• Prepare CFRs for eventual stress and emotional response
• Prepare CFRs regarding how to handle eventual barriers to arriving to the scene
• Include only instructions to accessible AEDs in the app
• Give precise instructions in the app of how to get to the AED and to the scene
• Include some information about the victim in the app

When arriving at the scene

• Prepare CFRs regarding how to handle that emergency medical services may arrive first
• Prepare CFRs regarding how to handle the victim’s family and the unfamiliar environment, for example the feeling of being in someone’s home

During the resuscitation

• Prepare the CFRs regarding how to handle risks of contagion
• Prepare CFRs regarding eventual ethical issues
• Prepare CFRs regarding how to handle eventual conditions with other needs than CPR
• Provide possibilities for contact with dispatch service during the mission

Post-resuscitation

• Include discussions during education about the importance of talking with someone about the experience after the mission
• Provide possibilities for professional debriefing
• Provide possibilities for emotional support through the app
• Provide possibilities for victims’ family to get in touch with the CFR

AED; Automated External Defibrillator, CFR; Community First Responder (Smartphone-based activated), CPR; Cardiopulmonary resuscitation.

## Discussion

The findings in this interview study illustrate that the CFRs had a profound wish to help and were motivated by the importance of the mission. However, they simultaneously feared the situation and were not always prepared for their own reaction in the emergency when they were dispatched. Although the completion of CPR training gave them the skills and confidence that enabled them to act constructively, they faced unforeseen challenges, ethical dilemmas and emotional reactions. A European consensus conference^14^ recently concluded that training, preferably face-to-face simulation-based training, is fundamental for CFRs, but it is still uncertain which aspects other than CPR should be included. Our findings indicated a need to extend the preparation of CFR volunteers to include not only the practical skills of CPR but also training with the smartphone app and preparation related to psychological stress, risks of contagion, interactions with the victim’s family, other conditions and ethical dilemmas that they might face. Providing space for such discussions and other scenarios might promote wellbeing among CFRs. Additionally, for CFRs who are healthcare workers, it seems to be valuable to discuss the feeling of not being in the usual work role with access to information and materials.

According to the CFRs, essential actions were performed in collaboration, and most interactions were well functioning, which is consistent with other studies.^28^ However, AEDs that were located too far away or that were not accessible out of business hours were revealed to be a concern, which has also been highlighted by others.^29^ To eliminate avoidable delays, instructions in the app should only direct CFRs to accessible AEDs that are nearby. Preferably, CPR training participants could be encouraged to familiarize themselves with the locations of and eventual periods of restricted access to AEDs in their neighbourhoods. Additionally, more around-the-clock accessible public AEDs would be beneficial.

The app in the CFR system used by responders in the present study provided an address of the OCHA and a map but no opportunity for further contact with the dispatch service, which the informants perceived as frustrating. Several other CFR systems allow for communication between the CFR and the dispatch service, and some even provide videoconferencing.^16^ Communication between responders and dispatch services can reduce stress levels.^14^ Hence, this would be a beneficial function to include in future app development.

Participating as a CFR can be emotionally stressful^30^ and can even induce severe psychological impacts.^31^ In a recent study in Denmark, a Scandinavian country similar to Sweden, 22 of 1,621 (1.4%) CFRs reported a severe impact, and three of them were in need of professional follow-up.^11^ However, the incidence of distress varies between studies, probably due to contextual and methodological differences, and higher as well as lower incidences have also been reported.16., 31., 32., 33. In line with the results of Kragh et al.,^32^ the lay responders in our study seemed more affected than healthcare worker responders. Emotional reactions were mainly coped with through the support of family and friends, which is consistent with other findings.31., 34. Further investigation is required to determine whether training could help CFRs deal with emotional reactions and frustration.

A need for feedback and debriefing opportunities concerning the experienced situation was indicated, preferably offered in the smartphone app. Having the opportunity to share experiences is a supportive action.32., 35. Offering professional debriefing could complement support received from family and friends and, as Palsgaard Møller et al.^35^ highlighted, could contribute to CFRs’ confidence in their skills. Feedback that does not breach confidentiality could also give the responders important reassurance that they had given the victim a better chance, regardless of the actual outcome and thereby help them to manage the aftermath.^35^ Additionally, the CFRs highlighted feelings of guilt and recurrent thoughts of the victim when they were unable to accept an alert. The need for support for this group of responders must also be considered to prevent distress or potential withdrawal from voluntary commitment.

In cardiac arrests time to CPR significantly affects the survival rate.5., 6., 7., 8. However, Coons^36^ reported that approximately 45% of bystanders would not perform CPR on a stranger. Utilizing CFRs enables quick assistance to bystanders who witness an OHCA incident, and it increases the possibility of someone intervening before emergency medical services arrive by up to 15%.11., 7. Hence, engaging CFRs seems to be beneficial for societies.

The CFRs in our study perceived their mission to be important, as they contributed to society with their abilities and engagement. Their engagement could be utilized in the recruitment of more volunteers. Additionally, CFR participation could be more actively promoted and advertised both to the public and in healthcare settings. According to Farmer et al.,^37^ engaging citizens as volunteers can lead to greater responsibility in society and increase the public’s knowledge about health services and supportive technologies. Other researchers illustrate similar findings and note that CFR engagement may be a pathway to a career in the health professions.28., 38. Arranging social gatherings where CFRs can exchange experiences and maintain their engagement in and contributions to society might thereby be a good investment.

The findings in this interview study illustrate the CFRs’ experiences of OHCA alerts, providing insights into the potential for improvement regarding the CFR system, CPR training and aftermath support. To fully utilize CFRs as a resource, the gained insights can be used by system developers as well as healthcare service providers that adopt such services, and they can be considered in the further development of CPR training programs.

### Limitations and strengths

This study was limited by a volunteer sample of CFRs (14 interviews), which must be considered when assessing transferability. However, the CFRs described a total of 31 OHCA alerts, and the last interviews did not reveal any new perspective, which indicates saturation and sufficient material to draw reliable conclusions.^39^ No volunteers withdrew from interview participation. Credibility was promoted by the experience of qualitative research methods and collaboration between the authors. Quotes from the interviews are presented in the results, offering the reader the opportunity to judge the interpretation and trustworthiness of the findings. The qualitative study design enabled an in-depth understanding of the phenomenon from the perspective of the involved responders. The study did not include quantification or claims of causality. Conclusions regarding potential improvements were based on the informants’ statements. Further investigation is required to determine whether the suggestions could help CFRs deal with OHCA alert situations. Additionally, the experiences of the victims, families and emergency medical service providers would be of interest to broaden the understanding of the phenomenon.

## Conclusions

This interview study provided insights into how CFRs alerted via smartphone perceive and cope with OHCA alert situations. The responders had a profound wish to help and were motivated by the importance of the mission. However, they simultaneously feared the situation and were not always prepared for their own reactions in the emergency when they were dispatched. Although the completion of CPR training and past experience gave them skills and confidence that enabled them to act constructively, they faced unforeseen challenges, ethical dilemmas and emotional reactions that might be eased by improvements in the CFR system and CPR training. The CFRs were proud of their efforts and were good ambassadors for the system. Better appreciation of their commitment, better preparation and the provision of support in the aftermath of an emergency seem to represent a good investment in societies’ efforts to bring quick help to distressed persons.

## Conflicts of interest

All authors declare no conflicts of interest.

## CRediT authorship contribution statement

**Marie-Louise Södersved Källestedt:** Conceptualization, Formal analysis, Methodology, Data curation, Project administration, Validation, Visualization, Writing – original draft, Writing – review & editing. **Harald Lindén:** Methodology, Data curation, Visualization, Writing – review & editing. **Petronella Bjurling-Sjöberg:** Conceptualization, Formal analysis, Methodology, Data curation, Project administration, Validation, Visualization, Writing – original draft, Writing – review & editing.
