# Supplementary material for: Smartphone activated community first responders’ experiences of out-of-hospital cardiac arrests alerts, a qualitative study
Source: Resusc Plus. 2022 May 18;10:100246. doi: 10.1016/j.resplu.2022.100246 (PMC9123264; doi:10.1016/j.resplu.2022.100246)
Supplement: Supplementary data 1 [file mmc1.docx]

Out-of-hospital cardiac arrest
(someone calls the alarm number)

Emergency medical services

Fire fighters
(in some areas)

Dispatch~~-~~centre sends out alerts to...

CFR system
(If between 7 a.m.- 11 p.m. and victim is older than 8 years)

National AED register is used to locate the nearest AED

The nearest 30 CFRs within the radius (1800 metres) are identified

Algorithm calculates the most efficient distribution of mission

Alert are sent in the smartphone app to select CFRs

CFR accepts mission

CFR declines mission *

Selected CFR instructed to pick up appointed AED and then go to the OHCA, map is provided *

Selected CFR instructed to go to OHCA, map is provided *

Goes to the OHCA site

Drop out

Pick up AED

Drop out

Contribute on site

* Evaluation sent by the system to CFR
(regardless of accepting or declining the alert)

AED; Automated External Defibrillator

CFR; Community First Responder (smartphone-alerted)

OHCA; Out-of-Hospital Cardiac Arrest
