# Supplementary material for: Smartphone activated community first responders’ experiences of out-of-hospital cardiac arrests alerts, a qualitative study
Source: Resusc Plus. 2022 May 18;10:100246. doi: 10.1016/j.resplu.2022.100246 (PMC9123264; doi:10.1016/j.resplu.2022.100246)
Supplement: Supplementary data 2 [file mmc2.docx]

**Appendix 2. Interview guide**

**Demographics**

- Gender?
- Age?
- In what region are you a citizen?
- What is your profession?
- When did you complete your last CPR training?
- How many OHCA alerts have you received through the CFR system?
- When was your last CFR mission?

**Opening question**

- Can you please tell me about when you were called out as a CFR?

**Focus areas and potential probe questions**(Probes to be utilized if the area of interest is not covered by the informant’s spontaneous answer to the opening question)

**Area 1: Training and previous experience in CPR.**

- In what way were you prepared for a resuscitation situation?
- What knowledge from the training was most useful when receiving the OHCA alert?
- What did you lack from the training?
- What made you sign up as a CFR?
- Have you been in an OHCA situation before?

**Area 2: Reactions and actions when alerted.**

- What were your thoughts and reactions when you got the alert?
- How prepared did you feel as a CFR when you received the alert?
- What was the scenario at the scene of the cardiac arrest?
- How did you contribute?
- Have you received any information about the outcome?
- How did you feel afterwards?
- Have you needed any emotional support afterwards?
- What support would you have wanted?

**Area 3: Thoughts about being a CFR.**

- Are you still signed up as a CFR?
- Would you participate if you were alerted again?
- What contributes to your decision to be a CFR?
